# Acknowledgement to Reviewers

**DOI:** 10.1111/bpa.13139

**Published:** 2023-01-12

A thank you to our reviewers.

The editors at *Brain Pathology* would like to extend their gratitude to those who have provided their time and energy to review manuscripts for our journal over the last year. We are well aware that our journal can only exist thanks to your concerted efforts to provide concise, accurate, and thoughtful reviews. Below is a list of all of you who completed at least one review, and agreed to have your name published. We also thank those reviewers who choose not to have their names published.

Abassi, Zaid

Aktas, Gulali

Alafuzoff, Irina

Alexandrescu, Sanda

Amtul, Zareen

Bai, Hua

Bielle, Franck

Bieniek, Kevin

Cairns, Nigel

Capper, David

Carare, Roxana

Chang, Che‐Feng

Coppi, Elisabetta

Dahiya, Sonika

Darkwah Oppong, Marvin

Del Bigio, Marc

Diamandis, Phedias

Ding, Yi

Donato, Rosario

Eftekhapour, Eftekar

ElAli, Ayman

Erny, Daniel

Ferrer, Isidre

Fineschi, V

Fonseca, Ana Catarina

Friedrich, Christoph M

Gao, Yanqin

Gatto, Noemi

Guo, Jifeng

Han, Ying

Horbinski, Craig

Hu, Xue‐Yu

Irwin, David

Jiang, Nan

Kalaria, Raj

Kamasawa, Naomi

Kanat, Ayhan

Khodosevich, Konstantin

Kilic, E

Kovacs, Gabor

Latimer, Caitlin

Lattanzi, Simona

Marbacher, Serge

Martinez‐Marcos, Alino

Matute, Carlos

Mawrin, Christian

Miners, James

Mishra, Nishant

Miyata, Hajime

Möller, Harald

Morales, Rodrigo

Mueller, Norbert

Nakayama, Kazuhisa

Nauen, David

Neumann, Manuela

Park, Sung‐Hye

Paula‐Lima, Andrea

Pekmezci, Melike

Persidsky, Yuri

Piao, Yueshan

Pinho, João

Pinto, Luisa

Pollack, Saskia

Popa‐Wagner, Aurel

Pozzi, Silvia

Priller, Josef

Sarnat, Harvey

Sepulveda‐Falla, Diego

Shao, Anwen

Sinha, Namita

Sone, Daichi

Sreedharan, Jemeen

Stewart, Tessandra

Stewart, William

Stojanov, Dragan

Sun, Dianjianyi

Tapias, Victor

Theil, Thomas

Thom, M

Vaubel, Rachael

Vitte, Jeremie

Volbers, Bastian

Walker, Chandler

Wang, Jian

Wei, Zheng

Whitehead, Shawn

Wittenbecher, Clemens

Woo, Jung A

Wrede, Karsten

Wu, Di

Wu, Qihui

Yip, Stephen

Youssef, Sameh

Zhang, Shu

Zhang, Xiuming

Zhao, Cunyou

Zhou, Zhenhua

